# An Internal-State-Variable-Based Continuous Dynamic Recrystallization Model for Thermally Deformed TC18 Alloy

**DOI:** 10.3390/ma17164026

**Published:** 2024-08-13

**Authors:** Gui-Cheng Wu, Yong-Cheng Lin, Miao Wan, Ning-Fu Zeng, Song Zhang, Hui-Jie Zhang, Ming-Song Chen, Yu-Qiang Jiang

**Affiliations:** 1 School of Mechanical and Electrical Engineering, Central South University, Changsha 410083, China; 2 State Key Laboratory of Precision Manufacturing for Extreme Service Performance, Changsha 410083, China; 3 Light Alloy Research Institute, Central South University, Changsha 410083, China; 4 School of Materials Science and Engineering, East China Jiaotong University, Nanchang 330013, China

**Keywords:** internal state variables, continuous dynamic recrystallization, thermomechanical processing, microstructure, TC18 alloy

## Abstract

Continuous dynamic recrystallization (CDRX) is widely acknowledged to occur during hot forming and plays a significant role in microstructure development in alloys with moderate to high stacking fault energy. In this work, the flow stress and CDRX behaviors of the TC18 alloy subjected to hot deformation across a wide range of processing conditions are studied. It is observed that deformation leads to the formation of new low-angle grain boundaries (LAGBs). Subgrains rotate by absorbing dislocations, resulting in an increase in LAGB misorientation and the transition of some LAGBs into high-angle grain boundaries (HAGBs). The HAGBs migrate within the material, assimilating the (sub)grain boundaries. Subsequently, an internal state variable (ISV)-based CDRX model is developed, incorporating parameters such as the dislocation density, adiabatic temperature rise, subgrain rotation, LAGB area, HAGB area, and LAGB misorientation angle distribution. The values of the correlation coefficient (R), relative average absolute error (RAAE), and root-mean-square error (RMSE) between the anticipated true stress and measured stress are 0.989, 6.69%, and 4.78 MPa, respectively. The predicted outcomes demonstrate good agreement with experimental findings. The evolving trends of the subgrain boundary area under various conditions are quantitatively analyzed by assessing the changes in dynamic recovery (DRV)-eliminated dislocations and misorientation angles. Moreover, the ISV-based model accurately predicts the decreases in grain and crystallite sizes with higher strain rates and lower temperatures. The projected outcomes also indicate a transition from a stable and coarse-grained microstructure to a continuously recrystallized substructure.

## 1. Introduction

Thermomechanical processing plays a vital role in shaping and structuring alloy components with specific microstructures and properties [1,2]. During thermal deformation, dynamic recrystallization (DRX) and dynamic recovery (DRV) commonly take place. DRX is preferred for its effective microstructure adjustment, especially in grain refinement [3]. The refinement of β grains substantially improves the ductility and fatigue life of the final components. Additionally, this process can diminish the micro-texture and enhance the efficacy of ultrasonic flaw detection. Generally, DRX mechanisms include discontinuous dynamic recrystallization (DDRX), continuous dynamic recrystallization (CDRX), and geometric dynamic recrystallization (GDRX) [4,5,6]. One of these DRX mechanisms usually dominates under specific conditions.

DDRX, characterized by nucleation and growth at grain boundaries, typically occurs in materials with low stacking fault energy (SFE). Ti alloys with moderate to high SFE values of 0.31 J/m^2^ and 0.159 J/m^2^, corresponding to pure titanium and the addition of 5 wt.% aluminum, experience climbs and cross-slips of dislocations during thermal forming processes [7,8]. In such cases, the DRV mechanism can be easily activated to impede the accumulation of dislocations crucial for initiating DDRX during hot working. Following DRV, CDRX then becomes the primary mechanism in microstructure development [9]. GDRX occurs frequently during severe plastic deformation, where the strain often exceeds 3. This process entails the flattening of coarse grains to several times the size of subgrains, leading to a pinching effect at the serrated HAGBs, followed by the generation of new fine recrystallized grains [4,10].

In summary, the DDRX and GDRX mechanisms do not predominantly influence the microstructure development of Ti alloys in the β phase. Instead, the microstructure evolution during thermomechanical processing is characterized by the CDRX mechanism [11,12,13]. CDRX is commonly defined as a process where subgrains develop via DRV, leading to an increase in the misorientation angles of subgrain boundaries and ultimately resulting in the generation of new recrystallized grains. Unlike the DDRX mechanism, the key characteristics of CDRX can be summarized as follows [11,14,15]: (i) flow curves indicate the typical DRV behavior, starting with a steady state and culminating in a maximum point; (ii) subgrains are formed within the initial grains and transform into recrystallized grains by absorbing dislocations; (iii) the crystallite size decreases rapidly at moderate strains, but a fully recrystallized structure is expected at large strains (ε=7); (iv) a distinct preferred orientation emerges prior to complete recrystallization.

Modeling microstructure evolution is imperative for optimizing processing parameters, considering the high costs of experimental engineering and the constraints in idealizing the experiment process [16,17]. Several CDRX models have been proposed to address the complex variations in microstructure evolution compared to DDRX and GDRX behaviors. The pioneering GM model, introduced by Gourdet [11], focuses on understanding CDRX behaviors. This model principally addresses the following issues: (1) improving the dislocation density evolution model by incorporating work hardening (WH), DRV, and the migration of high-angle grain boundaries (HAGBs); (2) distinguishing DRV’s role in creating low-angle grain boundaries (LAGBs) and their transition into HAGBs; (3) expanding the internal state variables (ISVs) to include the mean dislocation density within subgrains or LAGBs, LAGB misorientation, crystallite size, and the proportions of LAGBs and HAGBs. The GM model can predict the crystallite size and misorientation angle distribution in a deformed structure but does not account for the LAGB energy, LAGB misorientation, strain rates, or (sub)grain size influencing subgrain rotation. Sun and Wu [8] further extended the GM model to include factors like the dislocation density and accumulated energy at subgrain boundaries to analyze subgrain rotation. The flow response and CDRX structure of the AA7075 alloy during thermal compression were adequately simulated, but the evolutionary trends of relative ISVs were not discussed. Based on the GM model, Li and Gu [18] developed a set of ISV formulas to describe viscoplastic flow behavior and microstructure evolution. The predicted saturated HAGB fraction and mean LAGB misorientation angle were in good agreement with experimental data. Tian and Chen [19] improved the GM model by incorporating the micron-level precipitate phase and grain boundary into the dislocation model. The effects of strain concentration and the enhancement of recrystallized grain formation near the grain boundary and precipitate phase are elucidated. Maizza [20] and Li [18] additionally investigated the effect of grain size on the WH part of the dislocation model, proposing that grain boundaries may assist in facilitating CDRX processes. Recently, Buzolin [21,22] modified the GM model to characterize the CDRX behaviors in a two-phase compressed Ti5553 alloy. Their research effectively forecasted load distribution, flow stress, and microstructural changes. Nonetheless, the effect of adiabatic heating was neglected. The rise in temperature due to the deformation work may affect the dislocation movement, deformation activation energy, grain size, and flow stress.

As previously mentioned, the microstructure evolution mechanism for a coarse and static recrystallized structure of a TC18 alloy subjected to thermal deformation is not well understood, and there is a lack of suitable ISV-based models addressing this issue. This study aims to develop ISV-based equations to understand the flow response and CDRX behaviors of the TC18 alloy during hot deformation across a wide range of processing conditions. This model takes into account factors including the dislocation density, adiabatic heat, LAGB formation, subgrain misorientation changes, HAGB and LAGB migration, and LAGB-to-HAGB conversion. The findings of this research can offer guidance and valuable insights for the systematic development of process designs for the primary hot working of coarse-grained Ti alloys.

## 2. Experiments

The TC18 alloy received was a wrought bar supplied by Hunan Goldsky Titanium Industry Technology Company in China. Its chemical composition (wt.) is 5.16% aluminum (Al), 4.92% molybdenum (Mo), 4.96% vanadium (V), 1.10% iron (Fe), and 0.98% chromium (Cr), with the remaining portion being titanium (Ti). The β-transus temperature was determined to be 1148 K [23]. To obtain a uniform and complete β-grain structure, a solution treatment was conducted at 1173 K for one hour before the compression test. Standard samples 8 mm in diameter and 12 mm in length were fabricated from the solution-treated alloy. High-temperature compression tests were carried out using the Gleeble 3500 machine at 1163 K, 1193 K, 1223 K, and 1253 K, with strain rates of 0.001, 0.01, 0.1, and 1 s^−1^. In order to minimize the effects of friction, tantalum foils were utilized between the samples and dies. Following deformation, immediate water quenching was executed to maintain the instantaneous microstructure.

After thermal compression, electron backscattered diffraction (EBSD) tests were performed. Electrochemical polishing was employed to prepare EBSD samples. An etching solution with 5 mL of perchloric acid (HClO_4_) and 95 mL of acetic acid (CH_3_COOH) was applied for a duration of 40 s at a voltage ranging from 30 to 40 volts. The EBSD analysis was performed utilizing a scanning electron microscope (JSM-7800 F) at an operating voltage of 20 kV. The data under examination were collected at intervals of 5 μm and analyzed utilizing OIM Analysis and the Channel 5 software.

## 3. Continuous Dynamic Recrystallization Behavior of TC18 Alloy

**Figure 1:**
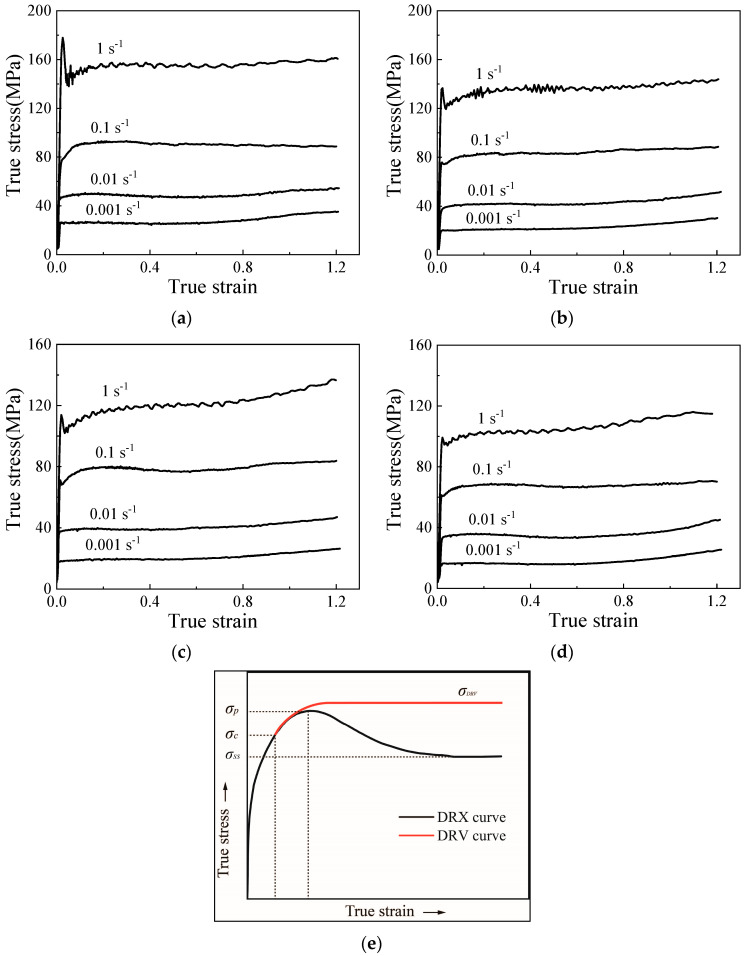
The flow curves of the TC18 alloy under specific conditions: (**a**) 1163 K; (**b**) 1193 K; (**c**) 1223 K; (**d**) 1253 K [23]. (**e**) The classical DRV and DRX flow curves.

The flow curves under different conditions (temperatures ranging between 1163 K and 1253 K and strain rates ranging between 0.001 and 1 s^−1^) are shown in Figure 1. The stress–strain curves climb rapidly at small strains due to the elastic deformation and WH effect. Subsequently, the peak stress is reached, after which a steady state emerges. There is no apparent decrease in stress, indicating that the DDRX behavior does not predominantly influence the thermal compression of the TC18 alloy. At a high strain rate of 1 s^−1^, a distinct discontinuous yielding phenomenon is observed, characterized by the stress–strain curves initially rising steeply and then dropping sharply. This phenomenon is attributed to the difference in critical resolved shear stress for dislocation activation and glide, according to the kinetic theory [24,25].

Stress–strain curves at large strains (exceeding 0.9) exhibit a general upward trend, attributed to the friction on the upper and lower end faces of cylindrical samples. Figure 1e displays the typical DRV and DRX flow curves. DRX-type curves show a significant decrease after reaching the peak stress ($\sigma_{p}$), followed by consistent stress at equilibrium observed at relatively large strains. Conversely, the DRV-type curves transition directly to the steady-state stage after the peak stress [4,26]. Based on the flow-curve characteristics of the TC18 alloy, the microstructure evolution during thermal forming is controlled by the DRV mechanism rather than the DDRX mechanism. A significant number of LAGBs are formed via DRV, increasing the misorientation angle through dislocation condensation at the boundaries, ultimately resulting in the formation of DRX grains. Essentially, CDRX is an extension of the ongoing process of DRV.

**Figure 2:**
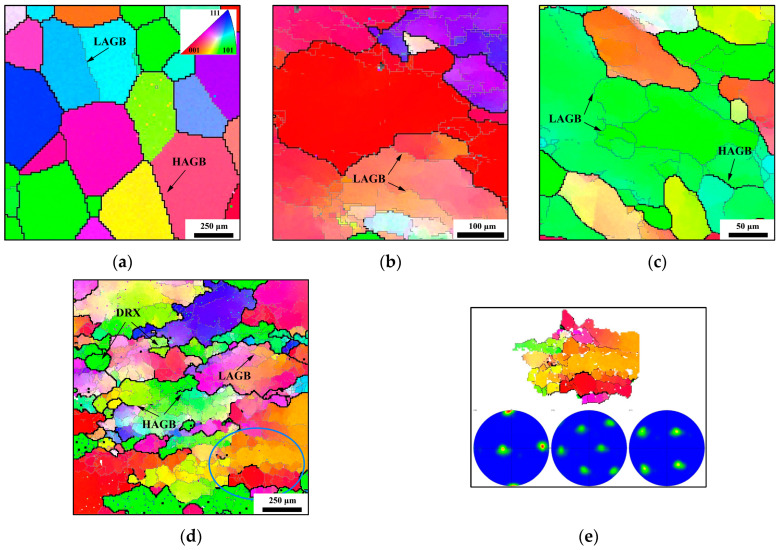
EBSD maps (the bold black lines are for HAGBs, while the thin gray lines are for LAGBs): (**a**) the initial structure; (**b**) 1163 K, 0.001 s^−1^, 0.3 (strain); (**c**) 1163 K, 0.001 s^−1^, 0.6 (strain); (**d**) 1163 K, 0.001 s^−1^, 1.2 (strain) [23]; (**e**) a pole figure of the blue elliptical area in Figure 2d.

Figure 2 illustrates the inverse pole figures (IPFs) of the initial and strained samples. LAGBs are represented by thin gray lines, and HAGBs are indicated by thick black lines. Subgrain boundaries with a misorientation angle below 2° were excluded due to the resolution limit of the testing machine. The same 2° threshold was used for the ISV models in Section 4. The EBSD map of the initial sample (Figure 2a) displays large, evenly distributed β grains without the presence of an α phase. In spite of the solution treatment in the β-phase domain, there are still a small number of LAGBs, with no evidence of tortuous or intermittent LAGBs within the grain interior.

The microstructures of the TC18 alloy strained at 1163 K and 0.001 s^−1^ with varying compression amounts are illustrated in Figure 2b–d. At the low strain (0.3), the LAGBs were progressively formed through dislocation entanglement, combination, and condensation, as denoted by the slender arrows in Figure 2b. As the strain increases, the condensation of dislocations at the boundaries increases the misorientation angle of LAGBs, giving rise to the progressive transformation of LAGBs into HAGBs. The appearance of discontinuous HAGBs within grain interior, as displayed in Figure 2c, confirms this observation. Upon reaching a compression level of 70%, as illustrated in Figure 2d, more HAGBs are observed within the grain interior. Some subgrains are enclosed by both LAGBs and HAGBs, and the noticeable color gradient inside the original grain interior indicates a rise in misorientation and the formation of numerous subgrains. Additionally, recrystallized grains are visible near the boundaries or within the original grain interior. The pole figure extracted from Figure 2d is displayed in Figure 2e. Despite the visible color gradient, the orientational information given by the pole figure is concentrated, implying the clear development of crystallographic texture.

**Figure 3:**
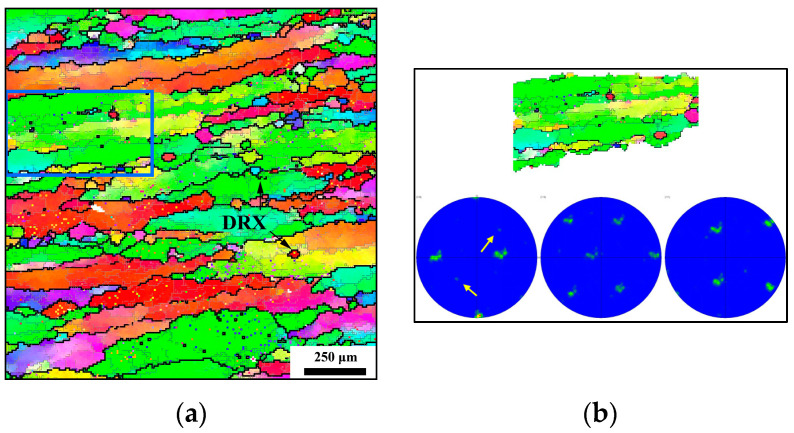
EBSD maps at (**a**) 1223 K, 0.01 s^−1^, 1.2 (strain) [23]; (**b**) pole figure of blue rectangular area in Figure 3a.

In contrast to Figure 2d (1163 K-0.001 s^−1^-1.2), a more distinct elongated β grain is observed in Figure 3a at 1223 K, 0.01 s^−1^, and 70% compression. The limited time, coupled with the lower strain rate of 0.001 s^−1^, hinders the significant migration of HAGBs, resulting in a flattened β-grain shape. Despite this, the zig-zagging and intermittent HAGBs are still present within the prior β grain and in its vicinity, signifying a considerable shift from LAGBs to HAGBs. Moreover, smaller recrystallized grains are evident compared to Figure 2d, with the dimensions of the DRX grain closely resembling those of a subgrain. Similar to Figure 2e, a blue rectangular area is delineated in Figure 3a, with the corresponding pole figure displayed in Figure 3b. The orientation of the majority of the rectangular region remains consistent, while the altered orientation of the recrystallized grains is highlighted by white arrows in Figure 3b.

**Figure 4:**
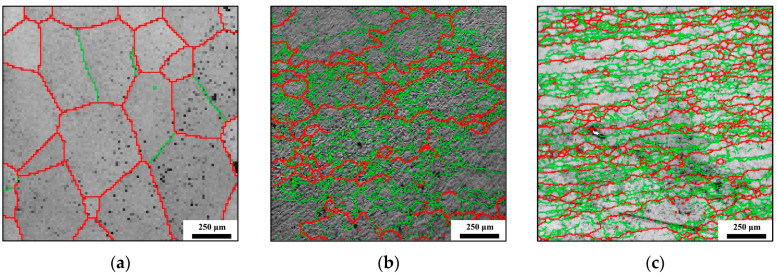
GB distribution maps (the bold red lines are for HAGBs, while the thin green lines are for LAGBs): (**a**) the initial structure; (**b**) 1163 K, 0.001 s^−1^, 1.2 (strain); (**c**) 1223 K, 0.01 s^−1^, 1.2 (strain).

To elucidate the distributions of HGABs and LAGBs more clearly, the boundary profiles of the original structure and the deformed structure are presented in Figure 4. During hot deformation, a significant presence of LAGBs and HAGBs is noted, alongside emerging recrystallized grains within the original β grain and its vicinity, with dynamically recrystallized (DRX) grain sizes comparable to those of subgrains. The microstructure features mentioned above are indicative of CDRX behavior [18].

Overall, an in-depth examination of the microstructure evolution and flow behavior of the TC18 alloy indicates that the CDRX behavior predominantly influences microstructural changes during thermoplastic forming instead of DDRX behavior [27,28]. In Section 4, a CDRX model based on ISVs will be proposed and validated, and then the projected outcomes will be rigorously examined and discussed.

## 4. Continuous Dynamic Recrystallization Model of TC18 Alloy

The results from the hot-deformation experiments and microstructure analysis, along with prior research, highlight the significant impact of processing parameters on the flow behavior and microstructural evolution of the TC18 alloy. In order to improve the understanding of the relationships among the processing variables, the mechanical response, and microstructural evolution, a series of equations based on intrinsic factors such as the dislocation density, (sub)grain size, HAGB/LAGB areas, distribution frequency of subgrain boundaries, and adiabatic temperature rise were formulated.

### 4.1. Modeling of Flow Behavior

According to the thermal activation theory, true stress can be divided into thermally activated stress and thermally independent stress:

(1) $$\sigma =\sigma_{ath}+\sigma_{th}$$

The thermally independent stress, $\sigma_{ath}$, characterizes the resistance from long-range obstacles, typically related to dislocation interactions and (sub)grain boundary strengthening. This can be expressed as follows [29]:

(2) $$\sigma_{ath}=\lambda_{1}M\alpha \mu b\sqrt{\rho }+\lambda_{2}M\alpha \mu bS_{HAGB}/2+\lambda_{3}M\alpha \mu bS_{LAGB}/2$$

where M is the Taylor coefficient (3.06), which is used to convert the resolved shear stress values across various slip systems into effective stress. $\lambda_{1}$, $\lambda_{2}$, and $\lambda_{3}$ are material constants. α is the proportional coefficient (0.5). ρ is the dislocation density. b is the amplitude of the Burgers vector ($2.86\times 10^{-10}$) [30]. μ is the shear modulus, which varies with temperature. The intrinsic correlation between the shear modulus of the TC18 alloy and temperature was calculated using JMatPro V7.0 software and can be expressed as follows: $\mu =(-0.0153T+21.8847)\times 10^{3}$.

The first term in Equation (2) accounts for the stress induced by dislocation interactions, which is proportional to the square root of the dislocation density. The second and third terms correspond to the strengthening mechanisms from the grain boundary and subgrain boundary, respectively. $S_{HAGB}$ and $S_{LAGB}$ represent the unit volume grain boundary area and subgrain boundary area, respectively. Thermal activation stress due to dislocation movement past barriers like lattice imperfections, alloying elements, and interstitial impurities depends on the temperature and strain rate. This stress is approximately equal to the material’s yield strength. This can be expressed as [31]

(3) $$\sigma_{th}=MB{\left(\frac{\dot{\varepsilon }}{{\dot{\varepsilon }}_{0}}\exp(-T/G)\right)}^{s}$$

where ${\dot{\varepsilon }}_{0}$ is the reference shear strain rate (1 s^−1^). B is the strength coefficient, relating to the solid solution strengthening alloy elements, and G and s are the material constants.

By substituting Equations (2) and (3) into Equation (1), the rheological stress can be described by

(4) $$\sigma =\lambda_{1}M\alpha \mu b\sqrt{\rho }+\lambda_{2}M\alpha \mu bS_{HAGB}/2+\lambda_{3}M\alpha \mu bS_{LAGB}/2+MB{\left(\frac{\dot{\varepsilon }}{{\dot{\varepsilon }}_{0}}\exp(-T/G)\right)}^{s}$$

**Figure 5:**
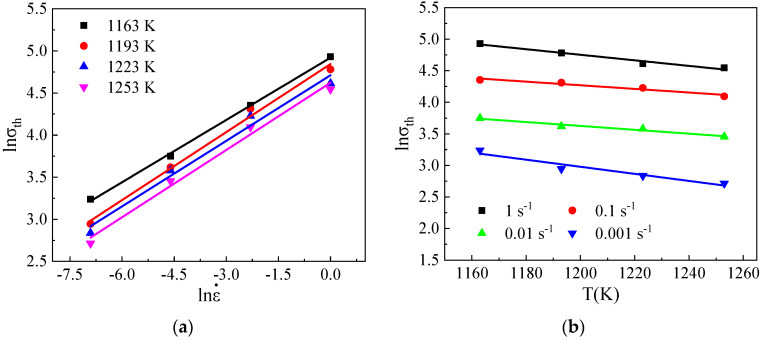
Plots of (**a**) $\ln\sigma_{th}-\ln\dot{\varepsilon }$ and (**b**) $\ln\sigma_{th}-T$ correlations.

By applying the logarithm to Equation (3), the expression $\ln\sigma_{th}=\ln M+\ln B+s\ln\left(\frac{\dot{\varepsilon }}{{\dot{\varepsilon }}_{0}}\right)+s(-T/G)$ can be derived. Substituting the yield stress and processing variables into Equation (3), the plots of $\ln\sigma_{th}-\ln\dot{\varepsilon }$ and $\ln\sigma_{th}-T$ can be obtained, as illustrated in Figure 5. The values of B, G, and s are finally determined to be 3406.26 MPa, 70.255 K, and 0.2604. Thermally activated stress can be described by $\sigma_{th}=10423.16{\left(\dot{\varepsilon }\exp(-T/70.255)\right)}^{0.2604}$.

### 4.2. Modeling of Microstructure Evolution

#### 4.2.1. Dislocation Density Model

During hot working, materials typically undergo WH and dynamic softening caused by recovery and recrystallization. These mechanisms coexist and compete, influencing the flow stress through the proliferation and annihilation of dislocations. Kocks [32] introduced a dislocation density model (KM model) to characterize the behaviors of WH and DRV:

(5) $$\frac{d\rho }{d\varepsilon }=k_{1}\sqrt{\rho }-k_{2}\rho$$

where $k_{1}$ denotes dislocation proliferation in WH, which is independent of temperature. $k_{2}$ represents dislocation elimination due to dislocation slip or/and climb during DRV and is characterized as a function of the temperature and strain rate [33]:

(6) $$k_{2}=k_{20}{\left(\dot{\varepsilon }\exp(\frac{Q_{k_{2}}}{RT})\right)}^{-q}$$

where $k_{20}$ and q are material constants. $Q_{k_{2}}$ is the deformation activation energy for the TC18 alloy and was determined to be 2.08 × 10^5^ J/mol by fitting the flow curves. R is the universal gas constant (8.3145 J/mol·K).

Meanwhile, DRX significantly reduces the dislocation density during the working process. The extended KM model accounts for the impact of recrystallization by incorporating a term that represents dislocation absorption in the volume displaced by HAGBs.

(7) $$d\rho =-\rho dV=-\rho S_{HAGB}vdt$$

where $S_{HAGB}$ is the area of HAGBs per unit volume, and v is the velocity at which grain boundaries migrate. The grain boundary migration speed results from the multiplication of the migration rate by the driving force [34]:

(8) $$v=M_{b}P$$

(9) $$M_{b}=\frac{b\delta D_{ob}}{K_{b}T}\exp(\frac{-Q_{b}}{RT})$$

(10) $$P=\rho \frac{\mu b^{2}}{2}$$

where δ, $D_{ob}$, $K_{b}$, $Q_{b}$, and $\mu b^{2}/2$ are the characteristic grain boundary thickness, grain boundary self-diffusion coefficient ($\delta D_{ob}$ is 5.4 × 10^−17^ m^3^/s), the Boltzmann constant (1.380 × 10^−23^ J/K), grain boundary self-diffusion activation energy (6.336 × 10^8^ J/mol), and line dislocation energy, respectively [35].

By combining Equations (7) and (5) and integrating strain hardening, dynamic recovery, and HAGB migration, the evolutionary equation of the dislocation density is obtained:

(11) $$d\rho =k_{1}\sqrt{\rho }d\varepsilon -k_{2}\rho d\varepsilon -k_{3}\rho S_{HAGB}vdt$$

where $k_{3}$ is the material constant.

#### 4.2.2. Subgrain Formation and Rotation Model

During hot forming in the β-phase domain, the β-phase structure undergoes dynamic recovery, leading to the formation of subgrains with a misorientation angle of less than 15°. Subgrain rotation is driven by stored energy, causing an increase in the misorientation angle of LAGBs. Once this angle exceeds 15°, subgrains transform into recrystallized grains. Thus, the existence of subgrains is essential for the initiation of CDRX.

Reference [11] demonstrates that the classical equation $\theta_{LAGB}=b/d_{0}$ (where $d_{0}$ is the distance between two dislocations) for an identical LAGB is satisfied. Consequently, the dislocation length per unit area can be calculated as $L_{\theta }=n\theta_{LAGB}/b$, where n represents the number of dislocation sets at grain boundaries. For example, n=2 refers to a symmetrical tilt grain boundary, and n=4 corresponds to a twist grain boundary [36]. Thus, the dislocation density, representing the dislocation length per unit volume, can be expressed as $\rho =n\theta_{LAGB}S_{LAGB}/b$. This correlation implies that an increase in $dS_{LAGB}$ with the mean misorientation of θ necessitates a dislocation consumption of dρ.

According to Equation (11), the dislocation density reduction caused by dynamic recovery is represented by $d\rho =k_{2}\rho d\varepsilon$. Some of the dislocations ($\alpha_{1}k_{2}\rho d\varepsilon$) are employed in forming LAGBs with the initial misorientation. Therefore, the increase in $S_{LAGB}$ can be expressed as [11]

(12) $$dS_{LAGB}^{1}=\frac{b}{n\theta_{0}}\alpha_{1}k_{2}\rho d\varepsilon$$

where $\theta_{0}$ is the initial angle (2°) for LAGBs.

The characteristic feature of newly formed subgrain boundaries is a low initial misorientation angle, defined as the critical threshold of 2°. In contrast to the nucleation and growth process observed in DDRX, the transformation of newly formed LAGBs into HAGBs is predominantly governed by subgrain rotation. The misorientation angle of LAGBs gradually increases until reaching the critical threshold of 15°. A portion of dislocations consumed in dynamic recovery are allocated to create LAGBs with the initial misorientation, while the rest are employed to increase the misorientation of subgrain boundaries, i.e.,

(13) $$d\theta_{LAGB}=(1-\alpha_{1})\frac{b}{nS_{LAGB}}k_{2}\rho d\varepsilon$$

Equation (13) indicates that the evolution of the orientation difference is influenced by the absorbed dislocations. Furthermore, the speed and amount of subgrain rotation are affected by subgrain size, initial grain size, subgrain misorientation, strain rates, and LAGB stored energy, as previously reported [37]. Considering these factors, a revised formula was derived to calculate variations in subgrain orientation.

(14) $$d\theta_{LAGB}=(1-\alpha_{1})\frac{b}{nS_{LAGB}}k_{2}\rho {\left(\frac{d_{ini}}{d_{ave}}\right)}^{\varphi_{1}}{\left(E_{LAGB}\right)}^{\varphi_{2}}{\dot{\varepsilon }}^{-c_{1}}d\varepsilon$$

where $d_{ini}$ and $d_{ave}$ are the initial and current average subgrain sizes, respectively. $\varphi_{1}$ and $\varphi_{2}$ are material constants. $E_{LAGB}$ is LAGB energy storage, which can be determined as follows [38]:

(15) $$E_{LAGB}=\frac{\mu b}{4\pi (1-\upsilon )}\theta_{LAGB}(1-\ln\frac{\theta_{LAGB}}{\theta_{m}})$$

where $\theta_{m}$ is the critical misorientation angle (15°), and υ is Poisson’s ratio (0.33).

Integrating Equations (14) and (15), the evolution of misorientation angle rotation can be written as

(16) $$d\theta_{LAGB}=(1-\alpha_{1})\frac{b}{nS_{LAGB}}k_{2}\rho {\left(\frac{d_{ini}}{d_{ave}}\right)}^{\varphi_{1}}{\left\{\frac{\mu b}{4\pi (1-\upsilon )}\theta_{LAGB}(1-\ln\frac{\theta_{LAGB}}{\theta_{m}})\right\}}^{\varphi_{2}}{\dot{\varepsilon }}^{-c_{1}}d\varepsilon$$

In the single-phase hot forming of the TC18 alloy, the average LAGB misorientation angle evolves consistently with the strain. Accordingly, the mean misorientation ($\bar{\theta }$) can be represented by

(17) $$\bar{\theta }=\frac{\int_{\theta_{0}}^{\theta_{m}}f(\theta_{LAGB})\theta_{LAGB}d\theta_{LAGB}}{f_{LAGB}}$$

where $f(\theta_{LAGB})$ is the orientation distribution function, and $f_{LAGB}$ is the area fraction of LAGBs.

Fresh LAGBs are continuously formed, while existing ones are also constantly consumed. Hence, it is essential to calculate the orientation distribution function of LAGBs for each incremental strain. Initially, the orientation of the newly formed LAGB distribution function can be expressed as

(18) $$f(\theta_{0})d\theta =\frac{dS_{LAGB}}{S}$$

where dθ is the orientation rotation amount of the LAGB with $\theta_{0}$ misorientation in the previous incremental step.

Additionally, as the strain increases, it becomes vital to assess the updated orientation distribution function, f(θ+dθ,ε+dε) [11]:

(19) $$f(\theta +d\theta ,\varepsilon +d\varepsilon )=f(\theta ,\varepsilon )(1+\frac{1}{D_{c}}\left(\frac{dD_{c}}{d\varepsilon }\right)d\varepsilon -dV)$$

where $D_{c}$ is the crystallite size.

#### 4.2.3. LAGB Area Evolution Model

During deformation, the primary factors influencing the variation in LAGB area are the formation of new LAGBs through the accumulation and rearrangement of dislocations, starting with a 2° misorientation angle. Conversely, subgrain boundaries gradually transition into HAGBs by absorbing dislocations, resulting in a decrease in the LAGB area. HAGB migration involves a specific volume and assimilates grain boundaries, while LAGB migration also absorbs a certain number of grain boundaries.

The calculation of the newly formed LAGB area with an initial orientation angle is presented in Equation (12). Throughout the CDRX process, subgrain rotation causes an increase in the misorientation angle of LAGBs. Some LAGBs transform into HAGBs, resulting in a decrease in the LAGB area [8].

(20) $$dS_{LAGB}^{2}=f(\theta_{c})Sd\theta$$

where $f(\theta_{c})$ and dθ represent the orientation distribution function and misorientation increment, respectively.

Additionally, HAGB migration also leads to a decrease in the LAGB area. The decreased LAGB area per unit of time can be calculated by

(21) $$dS_{LAGB}^{3}=\alpha_{2}S_{LAGB}S_{HAGB}M_{b}Pdt$$

where $\alpha_{2}$ is the material constant.

Simultaneously, the migration of LAGBs will sweep a certain volume and absorb other LAGBs [39]. These grain boundaries can be represented by $dS_{LAGB}^{4}=S_{LAGB}S_{LAGB}M_{LAGB}P_{LAGB}dt$. The migration rate of LAGBs increases rapidly as the misorientation angle rises, up to 15°, which can be expressed as

(22) $$M_{LAGB}=\beta_{1}{\left(\frac{\theta_{LAGB}}{\theta_{m}}\right)}^{\chi }M_{b}$$

where $\beta_{1}$ and χ are the material constants. A proportional correlation between $M_{b}$ and $M_{LAGB}$ with χ≈5.18 was utilized by Huang [40].

The main driving force for the migration of LAGBs, excluding second-phase particles, stems from the decreased storage energy within subgrains. Assuming that the subgrain is spherical and the driving force on its surface is evenly distributed, coupled with the Read–Shockley formula [38], the migration driving force can be formulated as follows:

(23) $$P_{LAGB}=(\frac{\theta_{LAGB}}{\theta_{m}})(1-\ln(\frac{\theta_{LAGB}}{\theta_{m}}))P$$

Therefore, the absorbed LAGB area can be expressed as

(24) $$dS_{LAGB}^{4}=\alpha_{3}{\left(S_{LAGB}\right)}^{2}{\left(\frac{\theta_{LAGB}}{\theta_{m}}\right)}^{\chi_{1}}(1-\ln(\frac{\theta_{LAGB}}{\theta_{m}}))M_{b}Pdt$$

where $\alpha_{3}$ is the material constant, and $\chi_{1}$ is 6.18.

The overall equation for the LAGB area evolution can be expressed as

(25) $$dS_{LAGB}=dS_{LAGB}^{1}-dS_{LAGB}^{2}-dS_{LAGB}^{3}-dS_{LAGB}^{4}$$

#### 4.2.4. HAGB Area Evolution Model

The evolution of the HAGB area mainly arises from two factors. The rotation of subgrains causes an increase in the orientation of LAGBs up to a critical value, transforming them into HAGBs. Simultaneously, the growth of recrystallized grains sweeps the existing HAGBs and generates new ones.

The increase in HAGB area due to subgrain rotation aligns with Equation (20).

(26) $$dS_{HAGB}^{1}=f(\theta_{c})Sd\theta$$

The movement of HAGBs results in the dissolution of both pre-existing and recently formed HAGBs. Therefore, the decrease in $S_{HAGB}$ due to the migration of HAGBs can be expressed as

(27) $$dS_{HAGB}^{2}=\beta_{2}S_{HAGB}S_{HAGB}M_{b}Pdt$$

where $\beta_{2}$ is the material constant.

Meanwhile, the migration of HAGBs resembles the expansion of recrystallized grains, facilitating the formation of new HAGBs. By approximating HAGBs as squares with an area of $S_{HAGB}$, the newly formed HAGB area through HAGB migration can be represented by $dS_{HAGB}=4\sqrt{S_{HAGB}}vdt$, subsequently revised to

(28) $$dS_{HAGB}^{3}=4\beta_{3}\sqrt{S_{HAGB}}M_{b}Pdt$$

where $\beta_{3}$ is the material constant.

The evolution of the HAGB area can be expressed as

(29) $$dS_{HAGB}=dS_{HAGB}^{1}-dS_{HAGB}^{2}+dS_{HAGB}^{3}$$

Furthermore, the total variation in the grain boundary area can be described as $dS=dS_{LAGB}+dS_{HAGB}$.

#### 4.2.5. Grain Size Model

Recrystallized grains gradually evolve from subgrains, leading to some crystallites being enclosed by HAGBs and LAGBs [19]. Stereological relationships are then employed to describe the grain size, subgrain size, and crystallite size. Throughout hot working, the subgrain size, grain size, and crystalline size all change as the strain increases, inversely correlated with the (sub)grain boundary area.

(30) $$D=2/S_{HAGB}$$

(31) $$d=2/S_{LAGB}$$

(32) $$D_{c}=2/S$$

#### 4.2.6. Temperature Rise Model

When a material undergoes plastic deformation due to external stress, deformation work is converted into distortion energy and thermal energy. The generation of deformation heat causes an increase in the compression specimen’s temperature, influencing the grain size, dislocation density, and deformation activation energy, thereby reducing the rheological stress of alloys. In the studies conducted by Zhang [41] and Semiatin [42], the formula for calculating adiabatic heating during hot working is as follows:

(33) $$\Delta T=\frac{\eta }{C_{v}d_{n}}\int_{0}^{\varepsilon }\sigma d\varepsilon$$

where ΔT is the adiabatic heating temperature rise, η is the efficiency of converting deformation energy into thermal energy (0.95), $C_{v}$ is the specific heat capacity (612 J/kg/℃), and $d_{n}$ is the material density (4.5 g/cm ^3^).

During high-temperature compression, the specimen exchanges heat with its surroundings. Lower strain rates lead to longer deformation times, resulting in increased heat conduction, radiation, and convection due to temperature differences. Thus, significant heat dissipation occurs. The temperature rise depends on the difference between deformation heat and heat loss. Equation (33) can be revised to

(34) $$dT=\frac{\eta }{C_{v}d_{n}}\int_{0}^{\varepsilon }\sigma d\varepsilon -\lambda^{\prime }\Delta Td\varepsilon$$

where $\lambda^{\prime }$ is the equivalent heat dissipation rate, and dT is the temperature rise caused by deformation. In an isothermal system, the current temperature T remains constant and is equivalent to the initial temperature $T_{0}$.

### 4.3. Identification of Material Constants

**Figure 6:**
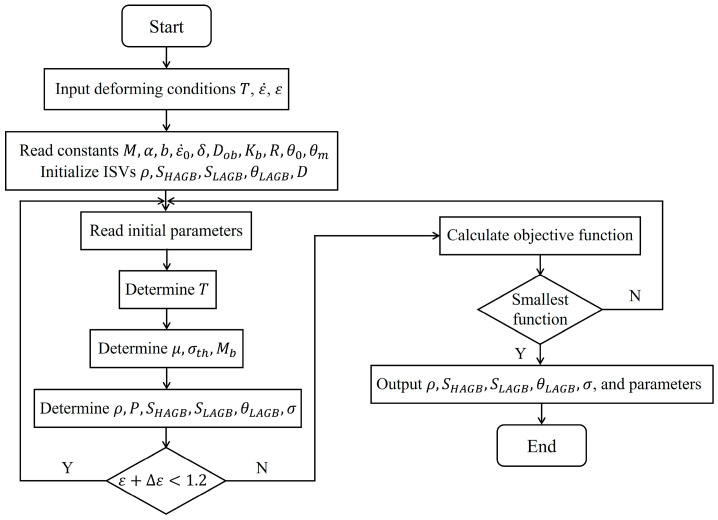
The solving flow of ISV-based models.

The ISV-based models incorporate multiple objective coefficients and variables, some of which are implicit. These models cannot be adequately solved by traditional linear fitting methods. A genetic algorithm method implemented in the MATLAB toolbox is utilized. Based on the experimental data, the following objective function is utilized:

(35) $$\left\{\begin{array}{l} f_{1}(x)=\sum_{i}w_{i}{\left(\frac{\sigma_{i}^{c}-\sigma_{i}^{e}}{\sigma_{i}^{e}}\right)}^{2}\\ f_{2}(x)=\sum_{j}w_{j}{\left(\frac{{\left(S_{HAGB}\right)}_{j}^{c}-{\left(S_{HAGB}\right)}_{j}^{e}}{{\left(S_{HAGB}\right)}_{j}^{e}}\right)}^{2}\\ f_{3}(x)=\sum_{k}w_{k}{\left(\frac{{\left(S_{LAGB}\right)}_{k}^{c}-{\left(S_{LAGB}\right)}_{k}^{e}}{{\left(S_{LAGB}\right)}_{k}^{e}}\right)}^{2} \end{array}\right.$$

where $f_{1}(x)$, $f_{2}(x)$, and $f_{3}(x)$ are residual errors of flow stress, HAGB area, and LAGB area, respectively. $x=[x_{1},x_{2},\ldots ,x_{h}]$ are the material constants of the TC18 alloy, h is the total number of material constants to be optimized, the superscripts c and e denote the computed and observed data, and $w_{i}$, $w_{j}$, and $w_{k}$ are weight coefficients. The objective formula is given by $f(x)=f_{1}(x)+f_{2}(x)+f_{3}(x)$. The ISV-based models are programmed in MATLAB, and the numerical optimization process is depicted in Figure 6.

The initial values of ISVs are set as follows: the HAGB area, LAGB area, and total GB area are 5713.7 m^−1^, 875.2 m^−1^, and 6588.9 m^−1^, respectively. The grain size, subgrain size, and crystalline size are 350 μm, 2285.2 μm, and 303.54 μm, respectively. The average misorientation angle of LAGBs is 7.38°. The initial dislocation density is 10^10^ m^−2^. The optimized material constants are detailed in Table 1.

### 4.4. Model Validation

**Figure 7:**
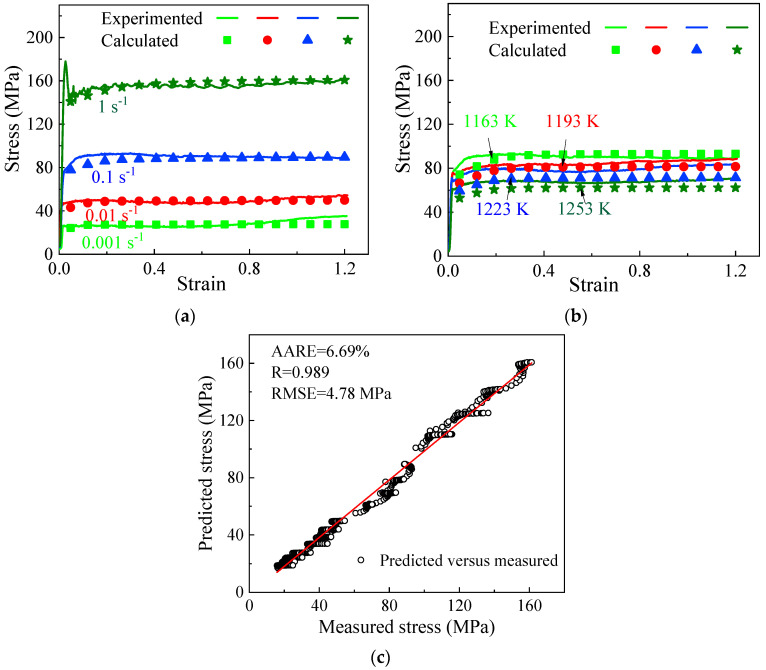
Comparisons between the calculated and experimental flow stresses at (**a**) 1163 K and (**b**) 0.1 s^−1^; (**c**) the correlation coefficient.

Figure 7 illustrates the discrepancies between the stress values obtained from experiments and those calculated. Solid lines represent experimental data, while symbols indicate calculated stresses. The anticipated true stress suitably aligns with the measured stress. Discrepancies between them can also be identified, possibly resulting from unconsidered microstructure evolution mechanisms such as DDRX and GDRX during hot deformation (Figure 3). These mechanisms lead to the creation of a considerable number of grain boundaries, thereby impeding the movement of dislocations. The predictive performance of the proposed ISV model can be evaluated using quantifiable metrics, including the correlation coefficient (R), relative average absolute error (RAAE), and root-mean-square error (RMSE) [43]. The values of R, RAAE, and RMSE are 0.989, 6.69%, and 4.78 MPa, respectively. Overall, the correlation findings suggest that the model can successfully predict stress levels in different deformation scenarios.

**Figure 8:**
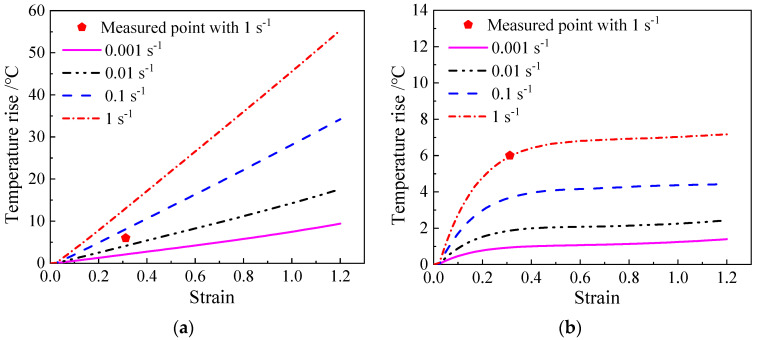
The temperature rise in the TC18 alloy compressed at 1253 K: (**a**) *λ*′ = 0, (**b**) *λ*′ = 0.163.

Figure 8 illustrates the comparison between the computed temperature increase in the TC18 alloy compressed at 1253 K with *λ*′ values of 0 and 0.163. The data point at 1 s^−1^ is depicted in Figure 8. Under the adiabatic condition (Figure 8a), the calculated temperature rise differs significantly from the measured result when *λ*′ is 0. However, the measured temperature rise closely matches the calculated data when the optimized *λ*′ value of 0.163 is used.

## 5. Results and Discussion

The MATLAB software was used to calculate the true stress as the strain increased, employing optimized material constants. Additionally, the intrinsic state variables, including the dislocation density, LAGB misorientation change, HAGB/LAGB areas, distribution frequency of LAGBs, and adiabatic temperature rise, were determined. The grain size and crystallite size were then calculated based on the stereological relationship described by Equations (30) and (32). Subsequently, the developed ISV model was utilized to predict the macroscopic mechanical response and microstructure development under various deformation conditions.

**Figure 9:**
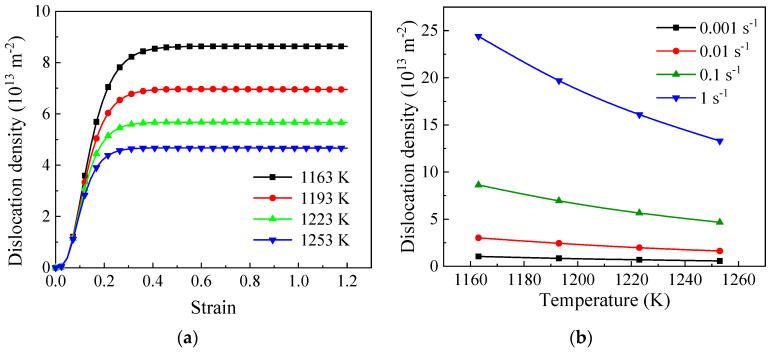
The evolutionary trends of dislocation density at (**a**) 0.1 s^−1^; (**b**) the various deformation parameters.

The variations in dislocation density trends at a strain rate of 0.1 s^−1^ are illustrated in Figure 9a. Like flow curves, dislocation densities increase until they peak and then stabilize. The influence of deformation parameters on the dislocation density is illustrated in Figure 9b. It is observed that the dislocation density increases with higher strain rates and lower temperatures. At low strain rates, there is enough time for dislocations to move, react, and reorganize, resulting in the formation of dislocation cells, also known as DRV [44,45]. In addition, the WH effect is weaker at low strain rates. Therefore, reinforced DRV decreases the dislocation density in tandem with weakened WH.

A higher deformation temperature typically results in a more extensive DRV process. During this phase, dislocations with opposite signs on the same slip plane cancel each other out, while dislocations on different slip planes undergo reorganization through cross-slipping and climbing to form LAGBs, thereby decreasing the dislocation density within grains. Elevated temperatures lead to increased atom vibrations as a result of the increased internal energy. This, in turn, facilitates the movement of dislocations through mechanisms such as gliding, climbing, and cross-slipping [46]. Furthermore, at elevated temperatures, CDRX occurs more frequently, and the accelerated migration of recrystallized GBs further eliminates a large number of dislocations.

**Figure 10:**
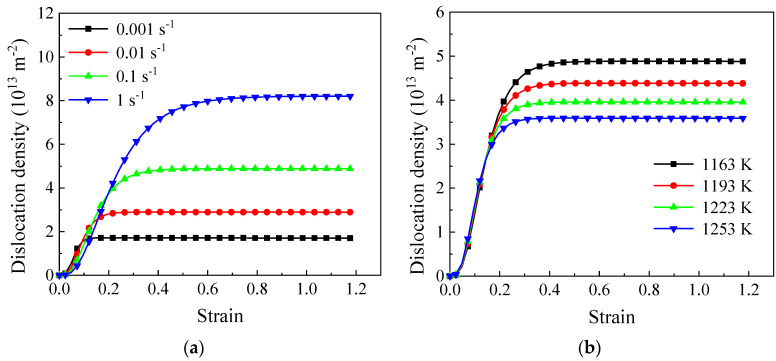
The evolutionary trends of dislocation density ($d\rho_{DRV}$) at (**a**) 1163 K; (**b**) 0.1 s^−1^.

**Figure 11:**
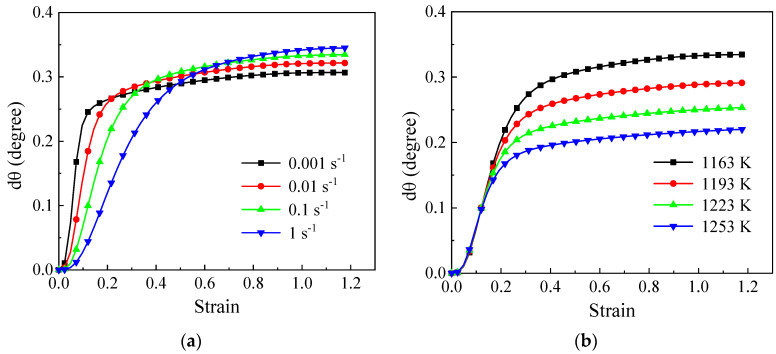
The evolutionary trends of misorientation of an LAGB (dθ) with an incipient misorientation angle of 2° at (**a**) 1163 K; (**b**) 0.1 s^−1^.

To improve the understanding of the microstructure evolution law during the thermoplastic forming of the TC18 alloy, the influences of various conditions on the changes in DRV-annihilated dislocations and the misorientation angle are investigated (Figure 10 and Figure 11). With an adequate duration at a low strain rate (0.001 s^−1^), DRV development is significant. Rapidly achieving a dynamic equilibrium between dislocation accumulation and condensation is feasible. As illustrated in Figure 10a, the DRV dislocation density ($d\rho_{DRV}$) stabilizes more quickly at lower strain rates. Additionally, an increased deformation rate and decreased temperature enhance the peak DRV dislocation density. ($d\rho_{DRV}$). The higher the DRV dislocation density, the greater the $S_{LAGB}$ (Equation (12)).

The variations in the misorientation angle, as determined by Equation (16), are influenced by the absorbed dislocations, subgrain size, strain rates, and stored energy in LAGBs. In this study, the changing trends of dθ within a specific LAGB starting with an initial misorientation angle of 2° are displayed in Figure 11. A more pronounced difference can be observed in comparison to DRV dislocation density curves, particularly in the early stage of deformation (ε<0.4). At low strain rates, dθ is larger, which can be attributed to the higher DRV dislocation density and lower strain rate specified in Equation (16). Lower deformation rates provide sufficient time for subgrain rotation. With increasing strain, dθ gradually rises with the higher dislocation density. Similar to the DRV dislocation density shown in Figure 10, dθ increases gradually. At lower strain rates, it stabilizes earlier, whereas at higher strain rates, it continues to rise. This trend can be elucidated by the higher DRV dislocation density and larger $S_{LAGB}$ area [19]. While the constant levels of dθ at various $\dot{\varepsilon }$ are relatively similar, the variation becomes more evident under varying temperature conditions, as illustrated in Figure 10.

**Figure 12:**
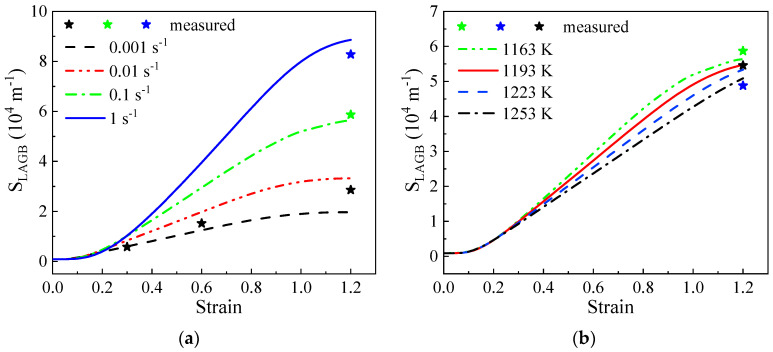
The evolutionary trends of the LAGB area at (**a**) 1163 K; (**b**) 0.1 s^−1^.

The evolution of $S_{LAGB}$ under different conditions is depicted in Figure 12. The solid line represents the predicted results under the corresponding conditions, while the measured data are shown as stars. The curves of $S_{LAGB}$ with varying strains are primarily categorized into three sections: the invariant, rapid growth, and steady-state sections. The initial invariant section (ε<0.1) mainly comprises the WH stage, during which plastic deformation significantly increases the dislocation density and enhances the dislocation reaction (Figure 9). However, due to the short duration and low dislocation density ($d\rho_{DRV}$), there is only a limited increase in the number of dislocations that form LAGBs and cause the misorientation of the original LAGBs (Figure 10 and Figure 11). Consequently, the curves of the LAGB area exhibit minimal changes throughout the WH stage.

In the second section, the curves of $S_{LAGB}$ show rapid growth from a previously stable state. With the progression of deformation, an increasing number of dislocations are eliminated through various dislocation movements, such as slipping, climbing, and cross-slipping, resulting in a decrease in dislocation accumulation and lattice distortion. Simultaneously, some DRV dislocations are utilized to form the initial LAGBs (Equation (12)), while others are involved in rotating subgrains (Equation (16)). Due to the coarse β grains, dislocations take longer to migrate to the subgrain boundaries. Consequently, this leads to a decrease in the degree of misorientation of an LAGB (dθ). Similarly, fewer LAGBs are transformed into HAGBs. Meanwhile, the LAGB area eliminated by HAGB and LAGB migration is relatively small. In summary, the predominant factor influencing the alteration in LAGB area among the four mechanisms is the increasing $dS_{LAGB}^{1}$.

The third section represents the transition of $S_{LAGB}$ from a phase of rapid growth to a phase of equilibrium. As deformation progresses in the second stage, there is a significant increase in the area of LAGBs, leading to a rapid decrease in subgrain size. This phenomenon improves the ability of subgrain boundaries to absorb dislocations. The changing trends in the misorientation of LAGBs (dθ) gradually reach the maximum (Figure 11), facilitating the conversion of LAGBs into HAGBs. Subsequently, similar to the evolution of the dislocation density, the LAGB area gradually reaches a state of saturation, attributed to the dynamic equilibrium among the generation of new LAGBs, the conversion of LAGBs into HAGBs, and the migration volume of LAGBs and HAGBs. According to the stereological relationship defined in Equation (31), the subgrain size can also be quantified.

In Figure 12, it is observed that at lower strain rates and higher temperatures, a smaller area of LAGBs is maintained due to the enhanced DRV effect. Moreover, the $S_{LAGB}$ curves at decreased strain rates reach the steady state sooner, indicating the swift achievement of a dynamic equilibrium between dislocation accumulation and annihilation. Furthermore, the simulation results (solid lines) demonstrate good agreement with the measured data (stars). The observed trends in the LAGB area align well with the findings by Buzolin et al. [21] and Su [47].

**Figure 13:**
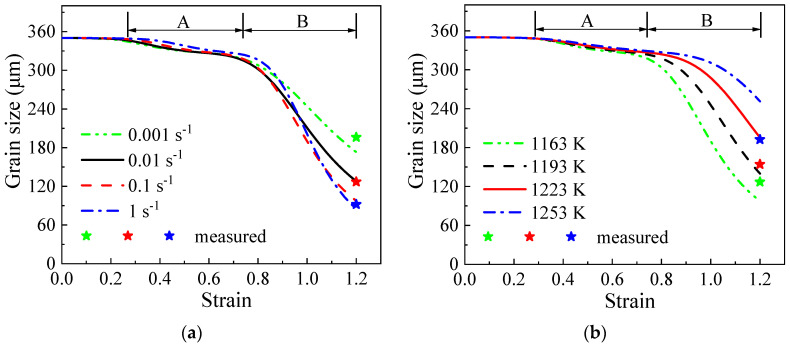
The evolutionary trends of grain size at (**a**) 1163 K; (**b**) 0.1 s^−1^.

**Figure 14:**
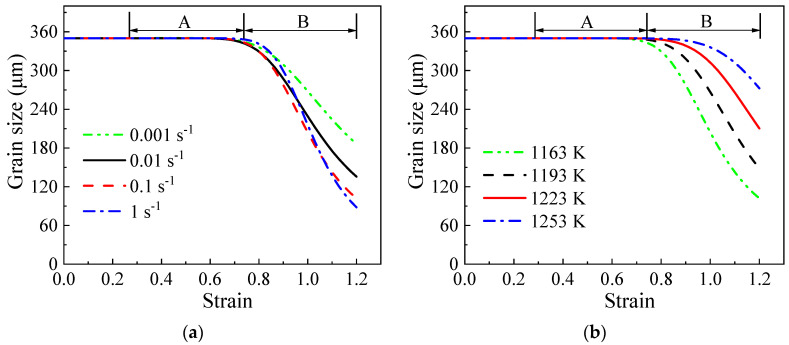
The evolutionary trends of grain size with the non-initial LAGB area at (**a**) 1163 K; (**b**) 0.1 s^−1^.

The variation curves of grain size can also be segmented into three distinct stages. The initial stage primarily consists of a section where the grain size remains almost constant. The second stage involves the transition of the grain size from a state of minimal change to a gradual decrease, identified as “A” in Figure 13. The third stage is characterized by a rapid decrease following the second phase, marked as “B”. The evolution of grain size can be elucidated by analyzing the changing trends of $S_{HAGB}$, as there is an inverse relationship between the two. The constant-phase one implies that there is no noticeable alteration in the HAGB area during the initial stages of the manufacturing process. The gradual decrease in the second phase indicates the solid transformation of LAGBs into HAGBs. It is crucial to highlight that this transformation stems from existing LAGBs rather than the newly formed LAGBs. The original LAGBs can be identified by the thin lines in Figure 4a, which account for 13% of the (sub)grain boundaries. For comparison, the change in grain size with the non-initial LAGB area is illustrated in Figure 14. It is evident that phases one and two merge with little variation, leading directly into phase three.

The grain size curves exhibit a rapid decrease with increasing strain (ε>0.7), suggesting a substantial transformation of newly formed LAGBs into HAGBs [28]. However, the curves only stabilize at the end of the deformation process (ε=1.2), reflecting the need for significant strain (above 7) to achieve complete recrystallization [21]. In industrial applications, a combination of different directions and multiple forging passes is recommended to achieve a refined and consistent grain structure [48]. Low temperatures and high strain rates lead to more LAGBs, promoting the transition of HAGBs and reducing the grain size. At 1163 K, the calculated grain sizes range from 172.9 to 84.9 μm across strain rates of 0.001 to 1 s^−1^. Similarly, at 0.1 s^−1^, the calculated grain sizes vary from 139.8 to 251.3 μm over temperatures from 1193 to 1253 K, showing good agreement with experimental data.

**Figure 15:**
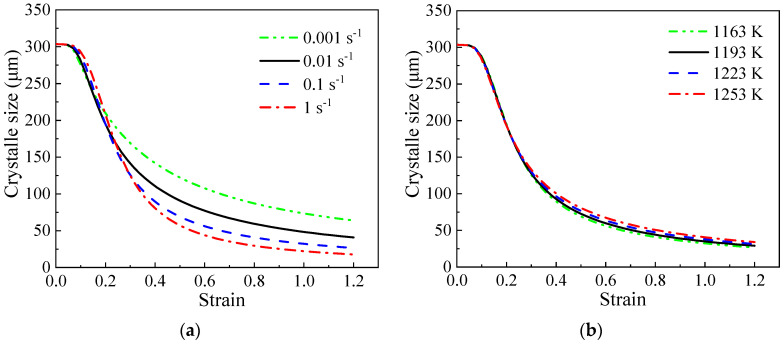
The evolutionary trends of crystallite size at (**a**) 1163 K; (**b**) 0.1 s^−1^.

During the CDRX process, DRV induces the formation of numerous LAGBs and the rotation of subgrains. The LAGBs gradually reach a dynamic equilibrium between consumption and generation, resulting in the coexistence of LAGBs and HAGBs throughout the entire straining process [49]. Consequently, following the suggestion by Gourdet [11], the average size of crystallites (Equation (32)), which are partially surrounded by LAGBs and partially by HAGBs, is utilized to characterize the refinement performance of initially coarse-grained alloys. Figure 15 illustrates the anticipated relationship between strain and crystallite size at varying deformation strain rates and temperatures. It is evident that the size of crystallites decreases notably with the applied strain. The refinement effectiveness is more noticeable at lower temperatures and higher strain rates. Additionally, compared to the deformation temperature, the average crystallite size exhibits greater sensitivity to strain rates. Similar to Figure 12, the curves depicting the crystallite size also show three distinct stages under all deformation conditions. In the initial stage, the curves remain stable due to the reduced density of dislocations and limited formation of LAGB areas. The second stage is characterized by a swift decline, indicating the formation of a significant number of LAGBs. The formation of new LAGBs is more prominent than the volume of (sub)grain boundary migration. Subsequently, the rate of decrease slows until reaching equilibrium.

## 6. Conclusions

This study quantitatively analyzed the mechanical properties and microstructure development predicted by the calibrated CDRX model. This computational methodology provides a satisfactory level of precision while requiring minimal computational resources. Consequently, it allows for modeling complex thermomechanical processes at an industrial scale. The primary findings are as follows:

1. The CDRX mechanism dominates the microstructure evolution in the TC18 alloy during β-phase deformation, converting the coarse recrystallized microstructure into a continuously recrystallized substructure. This transformation entails the formation of new LAGBs due to deformation; the rotation of subgrains through dislocation condensation, leading to an increase in the misorientation angle of LAGBs; the conversion of some LAGBs into HAGBs; and the migration of HAGBs, resulting in a decrease in the area occupied by both LAGBs and HAGBs.

2. An ISV-based CDRX model is proposed for the TC18 alloy, considering the dislocation density, subgrain rotation, LAGB area, HAGB area, LAGB misorientation angle distribution, and adiabatic heat. The proposed model introduces a term to account for strain rates in subgrain rotation. The values of R, RAAE, and RMSE between the calculated and measured true stresses are 0.989, 6.69%, and 4.78 MPa, respectively. The predicted results show good agreement with experimental data.

3. The ISV-based CDRX model was used to predict the evolving trends of the flow stress (σ), dislocation density (ρ and $d\rho_{DRV}$), LAGB misorientation (dθ), LAGB area ($S_{LAGB}$), grain size (D), and crystallite size ($D_{c}$) of the TC18 alloy under various thermoplastic conditions. The variation in grain size with the non-initial LAGB area was also comparatively analyzed. At 1163 K, the computed grain sizes vary between 172.9 μm and 84.9 μm when the strain rate ranges from 0.001 to 1 s⁻^1^.

## Figures and Tables

**Table 1:** The optimized material constants of the TC18 alloy.

$\lambda_{1}$ **(-)**	$\lambda_{2}$ **(-)**	$\lambda_{3}$ **(-)**	$k_{1}$ **(m^−1^)**	$k_{20}$ **(-)**	q **(-)**	$k_{3}$ **(m^−3^)**	$\alpha_{1}$ **(-)**	$\alpha_{2}$ **(-)**
0.67	22.3	29.1	2.19 × 108	1.08 × 103	0.2307	7.16 × 107	0.46	1.09 × 108
$\alpha_{3}$ **(-)**	$\beta_{2}$ **(-)**	$\beta_{3}$ **(-)**	$\varphi_{1}$ **(-)**	$\varphi_{2}$ **(-)**	$c_{1}$ **(-)**	s **(-)**	B **(MPa)**	G **(K)**
2.6 × 107	1.45 × 106	1.54 × 1010	1.09	0.25	0.23	0.2604	3.41 × 103	70.255

## Data Availability

Data will be made available on request.

## Nomenclature

σ, $\sigma_{ath}$, $\sigma_{th}$	flow stress, thermally independent stress, and thermal activation stress
ε, $\dot{\varepsilon }$	strain and strain rate
M, α, μ, b	Taylor coefficient, proportional coefficient, shear modulus, and Burgers vector
$S_{HAGB}$, $S_{LAGB}$, S	unit volume grain boundary area, subgrain boundary area, and sum of grain boundary and subgrain boundary areas
ρ	dislocation density
${\dot{\varepsilon }}_{0}$, B	reference shear strain rate and strength coefficient
$k_{2}$, $Q_{k_{2}}$	dislocation elimination coefficient and deformation activation energy
v, $M_{b}$, P	grain boundary migration velocity, migration rate, and driving force
δ, $D_{ob}$, $K_{b}$	characteristic grain boundary thickness, grain boundary self-diffusion coefficient, and Boltzmann constant
$Q_{b}$, R	grain boundary self-diffusion activation energy and universal gas constant
$d_{0}$, $\theta_{LAGB}$, $\theta_{0}$	distance between two dislocations, misorientation angle of LAGBs, and initial angle of LAGBs
$L_{\theta }$, n	dislocation length per unit area and number of dislocation sets at grain boundary
$d_{ini}$, $d_{ave}$	initial and current average subgrain sizes
$E_{LAGB}$, $\theta_{m}$, υ	LAGB energy storage, critical misorientation angle of LAGBs, and Poisson’s ratio
$\bar{\theta }$	mean misorientation of LAGBs
$f(\theta_{LAGB})$, $f_{LAGB}$	orientation distribution function and area fraction of LAGBs
D, d, $D_{c}$	grain size, subgrain size, and crystallite size
$M_{LAGB}$, $P_{LAGB}$	LAGB migration rate and driving force
ΔT, η, $C_{v}$, $d_{n}$	adiabatic heating temperature rise, efficiency of deformation energy conversion into thermal energy, specific heat capacity, and material density
dT, $T_{0}$, T, $\lambda^{\prime }$	temperature rise, initial temperature, current temperature, and equivalent heat dissipation rate
$dS_{LAGB}^{1}$, $dS_{LAGB}^{2}$, $dS_{LAGB}^{3}$, $dS_{LAGB}^{4}$	LAGB area with the initial misorientation, LAGB area transformed into HAGBs, LAGB area absorbed by HAGB migration, and LAGB area absorbed by LAGB migration
$dS_{HAGB}^{1}$, $dS_{HAGB}^{2}$, $dS_{HAGB}^{3}$	HAGB area due to subgrain rotation, HAGB area absorbed by HAGB migration, and new HAGB area formed by HAGB migration
$\lambda_{1}$, $\lambda_{2}$, $\lambda_{3}$, G, s, $k_{1}$, $k_{20}$, q, $k_{3}$, $\alpha_{1}$, $\varphi_{1}$, $\varphi_{2}$, $c_{1}$, $\alpha_{2}$, $\beta_{1}$, χ, $\alpha_{3}$, $\beta_{2}$, $\beta_{3}$	material constants
